# Exercise Training Enhances BDNF/TrkB Signaling Pathway and Inhibits Apoptosis in Diabetic Cerebral Cortex

**DOI:** 10.3390/ijms23126740

**Published:** 2022-06-16

**Authors:** Shiu-Min Cheng, Shin-Da Lee

**Affiliations:** 1 Department of Long-Term Care, National Quemoy University, Kinmen 892009, Taiwan; chengmin1988@gmail.com; 2 Department of Physical Therapy, Graduate Institute of Rehabilitation Science, China Medical University, Taichung 406040, Taiwan; 3 Department of Physical Therapy, Asia University, Taichung 413305, Taiwan; 4 School of Rehabilitation Medicine, Weifang Medical University, Weifang 261053, China

**Keywords:** apoptosis, BDNF, diabetes, exercise, neuroprotection

## Abstract

This study aimed to clarify the therapeutic effects of exercise training on neural BDNF/TrkB signaling and apoptotic pathways in diabetic cerebral cortex. Thirty-six male C57BL/6JNarl mice were randomly divided into three groups: control (CON-G), diabetic group (DM-G, 100 mg/kg streptozotocin, i.p.), and diabetic with exercise training group (DMEX-G, Swim training for 30 min/day, 5 days/week). After 12 weeks, H&E staining, TUNEL staining, and Western blotting were performed to detect the morphological changes, neural apoptosis, and protein levels in the cerebral cortex. The Bcl2, BclxL, and pBad were significant decreased in DM-G compared with CON-G, whereas they (excluded the Ras and pRaf1) were increased in DMEX-G. In addition, interstitial space and TUNEL(+) apoptotic cells found increased in DM-G with increases in Fas/FasL-mediated (FasL, Fas, FADD, cleaved-caspase-8, and cleaved-caspase-3) and mitochondria-initiated (tBid, Bax/Bcl2, Bak/BclxL, Bad, Apaf1, cytochrome *c*, and cleaved-caspase-9) apoptotic pathways. However, diabetes-induced neural apoptosis was less in DMEX-G than DM-G with observed raises in the BDNF/TrkB signaling pathway as well as decreases in Fas/FasL-mediated and mitochondria-initiated pathways. In conclusion, exercise training provided neuroprotective effects via enhanced neural BDNF/TrkB signaling pathway and prevent Fas/FasL-mediated and mitochondria-initiated apoptotic pathways in diabetic cerebral cortex.

## 1. Introduction

Diabetes is a chronic metabolic disease globally and the diabetic complications are commonly known for microvascular complications, including neuropathy, nephropathy, and retinopathy [1]. Diabetic encephalopathy is one of the diabetic complications and is characterized by slow, progressive changes in brain structures and disrupted cognitive function as impairments in learning and memory [2,3,4]. Recent evidence indicated that diabetes causes neural apoptosis and influences the central neural functions, such as cognitive dysfunctions [5,6,7,8]. To date, the precise pathogenesis of diabetic encephalopathy is complex and still not fully elucidated. Currently, the proportion of older adults and the length of life are increasing throughout the world. Studies indicated that diabetes is a risk factor for dementia, cognitive dysfunction, and Alzheimer’s disease, particularly with a higher risk among elderly diabetic patients [9,10]. Therefore, the mechanism of diabetes-induced neurodegeneration and encephalopathy urgently needs to be clarified.

Brain-derived neurotrophic factor (BDNF) was found widely in the central nervous system, including the hippocampus, cortex, hypothalamus, brainstem, and spinal cord [11,12,13]. BDNF has important functions in neuronal survival and growth, synaptic plasticity, neurogenesis, neural differentiation, and neuroprotection under hypoglycemia, neurotoxicity, and cerebral ischemia [12,13,14,15]. Functions of each of the neurotrophins are mediated through activation of one or more of the tropomyosin-related kinase (Trk) family of receptor tyrosine kinases (TrkA, TrkB, and TrkC) [16,17]. The Trk receptor-mediated intracellular signaling cascades, including the Ras/mitogen-activated protein kinases (MAPK, also known as extracellular signal regulated kinase (ERK)) protein kinase pathway, phosphatidylinositol3-kinase (PI3K)/protein kinase B (AKT) pathway, and phospholipase-C-γ (PLCγ)/diacylglycerol (DAG)/inositol 1,4,5 triphosphate (IP3) pathways [13]. BDNF binds to its high affinity TrkB receptor results in activation of three intracellular pathways, including PI3K/AKT, Ras/MEK/MAPK/ERK (MEK is a MAPK kinase that activates the MAPK), and PLCγ/DAG/IP3 pathways, leading to neural survival, neurogenesis, and neurite outgrowth [12,16,17]. Moreover, BDNF can activate the transcription factor cAMP response element binding protein (CREB) phosphorylation by the Ras/MAPK/ERK and PI3K/AKT pathways that regulate expression of genes involved in neuronal cell survival [11].

Previous studies reported that impairment of cognitive ability with decreased levels of BDNF, pTrkB, and Bcl2, and increased expressions of Bax and caspase-3 in hippocampus and cerebral cortex of streptozotocin-induced diabetic rat [18,19,20]. Moreover, low circulation BDNF levels accompany abnormal glucose metabolism and central decreased BDNF might be involved with type2 diabetes mellitus [21,22,23]. These data suggest that BDNF/TrkB signaling may play an indispensable role for preventing diabetes-induced neural apoptosis. However, the underlying mechanism of the BDNF/TrkB signaling and apoptotic pathways in the diabetic brain is not yet fully understood.

Acute exercise increased the serum BDNF level in healthy humans [24]. The hippocampal BDNF expression and cognitive function were improved in the rodents following exercise training [25,26]. The BDNF levels in the systemic circulation is elevated after prolonged exercise, possibly as a result of its release from the hippocampus and cortex since BDNF mRNA expression in the mouse hippocampus and cortex is increased in response to a single bout of exercise [27]. Generally, exercise is regarded as a beneficial nonpharmacological therapy for diabetes. Some studies demonstrated that exercise training increased the serum BDNF concentrations in diabetic patients and elevated hippocampal BDNF levels in the streptozotocin-induced diabetic animals [28,29]. Exercise training improved the learning and memory function, and the upregulation of the BDNF/TrkB/CREB signaling pathway in the hippocampus of streptozotocin-induced diabetic rats [30]. The above results suggest that exercise may be a better nonpharmacological therapy for increased BDNF levels and improved cognitive function in diabetes.

The current study was to understand the effects of exercise training on neural BDNF/TrkB signaling and apoptotic pathways in the diabetic cerebral cortex. We hypothesized that diabetes might predispose impaired neural BDNF/TrkB signaling and activated neural apoptotic pathways in diabetic cerebral cortex, which above effects may be reversed following exercise training.

## 2. Materials and Methods

### 2.1. Animals 

Thirty-six male C57BL/6JNarl mice (eight-week-old) were given access to tap water ad libitum and standard laboratory chow freely. The mice were housed in an enriched environment and maintained at a room temperature of 25 °C, with artificial 12 h day and night cycle. All experiments conformed to the protocol approved by the Institutional Animal Care and Use Committee of Asia University, Taichung, Taiwan (approval code: 98007). 

### 2.2. Diabetes Induction

The mice were injected with streptozotocin (100 mg/kg body weight, once daily, i.p.) dissolved in a sodium citrate buffer (pH 4.5). After the injections of streptozotocin, diabetes mellitus was considered as fasting blood glucose concentrations were maintained >11.1 mM or >200 mg/dL 48 h. Blood was sampled from the tail vein and the blood glucose concentrations were detected by Roche Accu Soft test strips.

### 2.3. Experimental Groups and Procedures

The animals were randomly separated into three groups (*n* = 12/each group): a control group (CON-G), wherein mice were injected with a sodium citrate buffer; the streptozotocin-induced diabetic group (DM-G); and the streptozotocin-induced diabetes with swimming exercise group (DMEX-G). One half of each group was selected randomly for pathological staining, while the other half was used for Western blotting analysis in each group.

The program for swimming training was modified following previous research [31]. In the first two weeks, the animals from DMEX-G swam for 15 min/day, 5 days/week, to begin their exercise training. Next, the duration of the training was prolonged to 20 min starting during the 3rd week and to 30 min during the 4th to 12th weeks. The device for swimming was a 60 × 90 × 50 cm water tub and the temperature of the water was maintained at 35 ± 1 °C. After the exercise training, animals were removed from the swimming pool, then gently dried with a towel and a hair dryer to avoid losing body temperature. All mice were euthanized 48 h after the end of experiments to avoid the acute effects of exercise.

### 2.4. Hematoxylin and Eosin (H&E) Stain

The cerebral cortex samples were soaked in 4% formalin at room temperature, then embedded in paraffin wax. The 3 μm thickness of paraffin sections were cut, stained with hematoxylin and eosin, and observed under a light microscope. The protocol of the stain process and observation were shown in our previous publication [32].

### 2.5. TUNEL Assay 

The sections of the paraffin-embedded cerebral cortex were deparaffinized using xylene and rehydrated, then covered in phosphate-buffered saline (PBS). H_2_O_2_ (2%) was added to inactivate endogenous peroxidases. Terminal deoxynucleotide transferase-mediated dUTP nick end labeling (TUNEL) assay and 4, 6-diamidino-2-phenylindole (DAPI) staining were performed as mentioned in our previous study [32]. Briefly, the tissue section was incubated with 20 μg/mL proteinase K, then washed in PBS and immersed in TUNEL reagent (apoptosis detection kit, Roche Applied Science). Brain sections were stained by using DAPI (Southern Biotech, Birmingham, AL, USA). For observation by fluorescence microscopy, the TUNEL(+) nuclei fluoresce green and the DAPI(+) nuclei fluoresce blue. 

### 2.6. Western Blot Analysis

After removing the brains from the mice, the cerebral cortex sample was isolated and immediately homogenizing in an ice-cold lysis buffer (100 mg tissue/1 mL buffer) and a protease inhibitor cocktail was added. The cerebral cortex samples were kept on ice to cool for 10 min, then centrifuged twice at 12,000× *g* for 40 min. Finally, supernatants were collected and stored at −70 °C for Western blot analysis. Lowry protein assay and Western blotting analysis were performed as previously reported with slight modification. [33]. The PVDF membrane was blocked in TBS buffer (containing 5% dry milk). Next, the PVDF membrane was incubated with indicated primary antibodies including Apaf1, AKT, pAKT(S473), PI3K, Bcl2, BclxL, pBad(S136), Bak, Cytochrome *c*, cleaved-caspase-9, cleaved-caspase-8, cleaved-caspase-3, CREB, pCREB(S133), ERK1/2, pERK1/2(T202/T204) (1:1000, Cell Signaling Technology Inc.), ATF1, pATF1(S63), Bad, FasL, Fas, FADD, GRB2, pGRB2(T452), GAB1, MEK1/2(I215), pMEK1/2(S218/S222), Ras, pRaf1(S338), RSK1/2/3, pRSK1(T359/S363), pTrkA(T490)/TrkB(T516) (1:1000, Novus Biologicals, USA), pPI3K(T508), α-tubulin (1:500, Santa Cruz Biotechnology), BDNF, Raf1 (1:1000, Millipore), and TrkB (1:1000, Thermo). A second antibody solution including goat anti-mouse IgG-HRP, goat anti-rabbit IgG-HRP, and donkey anti-goat IgG-HRP (1:5000, Santa Cruz Biotechnology) was used. The immunoblotted protein bands were then washed with TBS buffer. The protein bands were observed and quantified using a Fujifilm LAS-3000 chemiluminescence detection system (Fuji, Tokyo, Japan). 

### 2.7. Statistical Analysis

Statistical analysis was performed with the Kruskal–Wallis test and further analyzed with pre-planned comparison again negative control or positive control. TUNEL(+) apoptotic cells and protein expressions were compared among groups (CON-G, DM-G, and DMEX-G) using the Kruskal–Wallis test with preplanned contrast comparison against control group. CON-G acted as a negative control group for DM-G and DM-G acted as a non-treated control group for DMEX-G. The *p* value < 0.05 was considered statistically significant.

## 3. Results

### 3.1. Neural Histopathology and TUNEL(+) Apoptotic Cells

**Figure 1:**
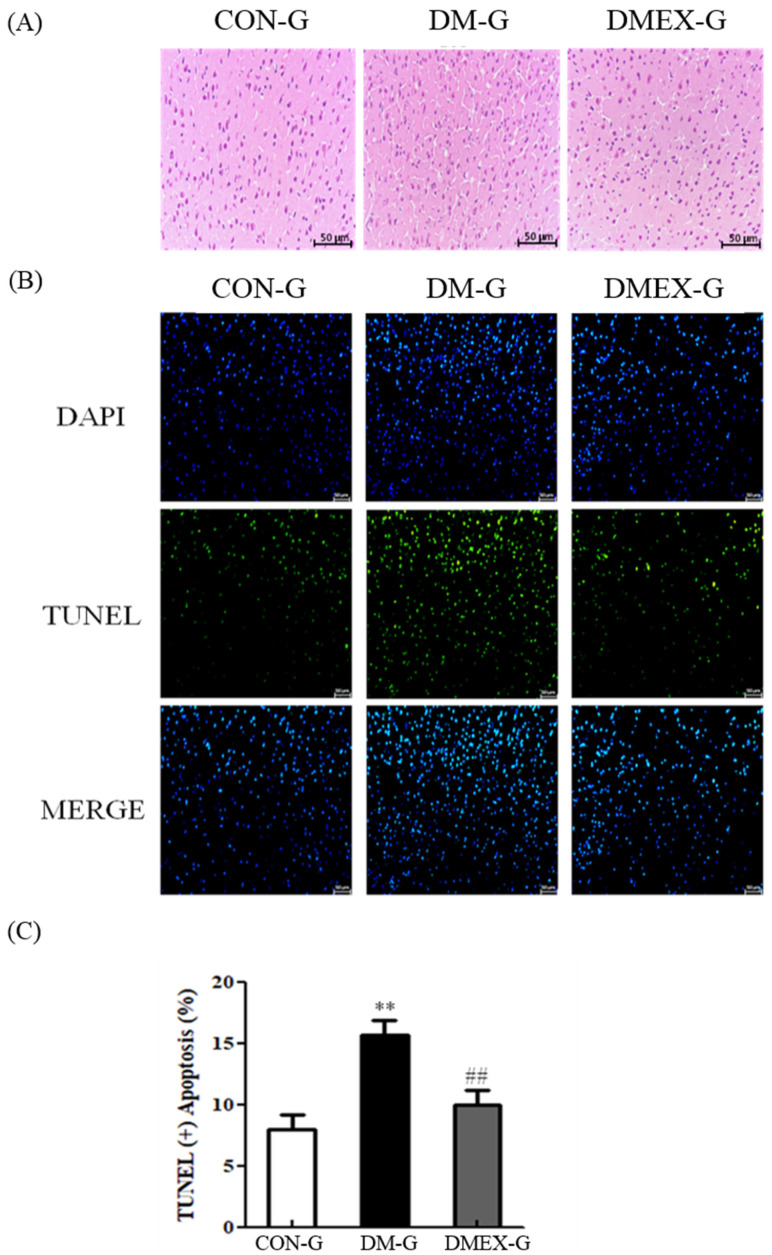
(**A**) Sections of cerebral cortex were stained with hematoxylin and eosin in the three groups (CON-G, DM-G and DMEX-G). Images of the brain section were magnified ×400. (**B**) The apoptotic cells of cerebral cortex in the three groups as stained with DAPI (top, blue spots) and TUNEL (bottom, green spots). (**C**) Percentage of TUNEL(+) cells relative to total cells are presented in bars. Data are expressed as mean ± SD (*n* = 3). ** *p* < 0.01 compared to CON-G, ## *p* < 0.01 compared to DM-G. CON-G, control group; DM-G, streptozotocin-induced diabetic group; DMEX-G, streptozotocin-induced diabetes with swimming exercise group.

To determine the changes of architecture and apoptosis in the diabetic cerebral cortex after swimming training, we used H&E staining and TUNEL assay. The cerebral cortex slices of CON-G displayed a normal architecture. In contrast with CON-G, interstitial spaces of DM-G exhibited enlargement, whereas these were attenuated in DMEX-G (Figure 1A). In addition, the cerebral cortex of DM-G showed significantly increased TUNEL(+) neural cells than those in CON-G. The number of TUNEL(+) neural cells in DMEX-G was significantly reduced compared to DM-G (Figure 1B,C).

### 3.2. Neural Extrinsic (Fas/FasL-Mediated) Apoptotic Pathway

**Figure 2:**
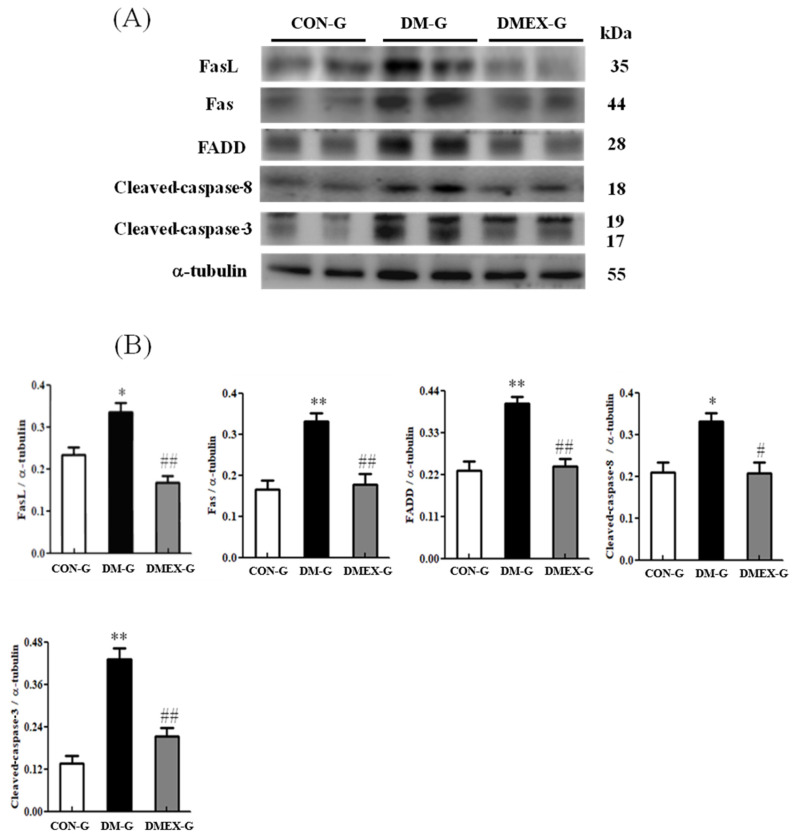
(**A**) Western blot analysis to determine the expressions of Fas ligand (FasL), Fas receptor (Fas), FADD, cleaved-caspase-8, and cleaved-caspase 3 in the cerebral cortex of CON-G, DM-G, and DMEX-G. (**B**) Relative protein quantification of FasL, Fas, FADD, cleaved-caspase-8, and cleaved-caspase 3 normalized to α-tubulin protein, respectively. Data are expressed as mean ± SD (*n* = 6). * *p* < 0.05, ** *p* < 0.01 compared to CON-G; # *p* < 0.05, ## *p* < 0.01 compared to DM-G. CON-G, control group; DM-G, streptozotocin-induced diabetic group; DMEX-G, streptozotocin-induced diabetes with swimming exercise group.

To identify the neural Fas/FasL-mediated apoptotic pathway in the diabetic cerebral cortex after swimming training, we determined the components of Fas/FasL-mediated apoptotic pathways in CON-G, DM-G, and DMEX-G. The expressions of FasL, Fas, FADD, cleaved-caspase-8, and cleaved-caspase-3 were significantly raised in DM-G compared to CON-G (Figure 2). However, the expressions of FasL, Fas, FADD, cleaved-caspase-8, and cleaved-caspase-3 in DMEX-G were significantly reduced compared to DM-G (Figure 2).

### 3.3. Neural Intrinsic (Mitochondria-Initiated) Apoptotic Pathway

**Figure 3:**
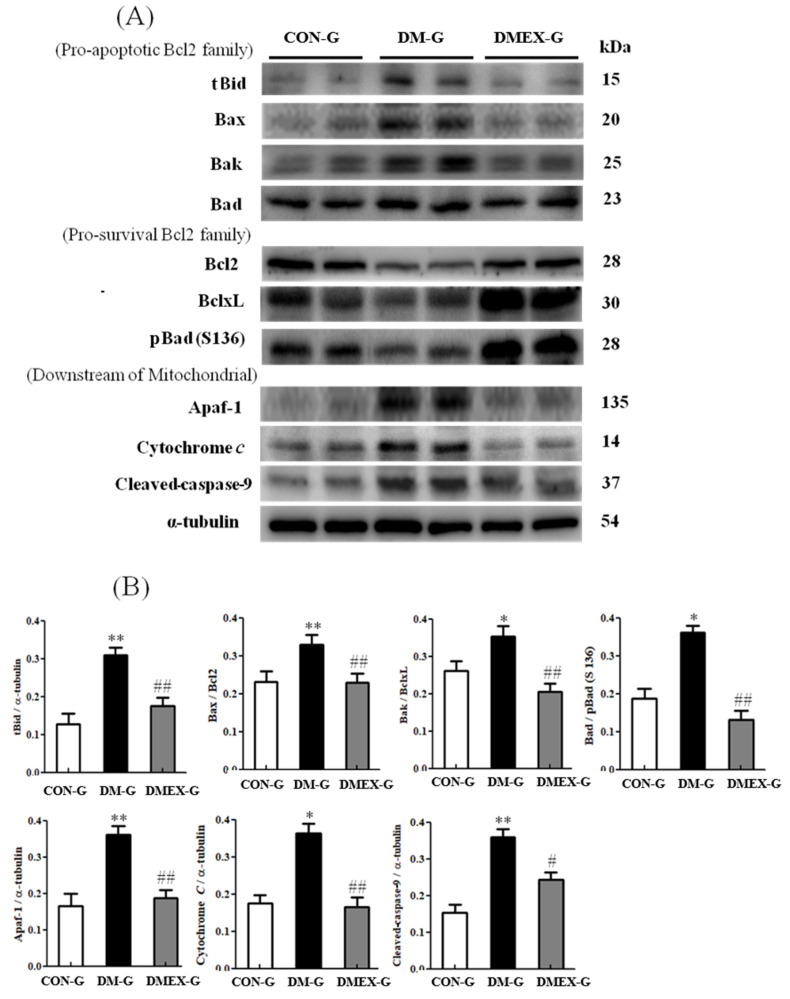
(**A**) Western blot analysis to determine the expressions of proapoptotic Bcl2-family (tBid, Bax, Bak and Bad), prosurvival Bcl2-family (Bcl2, BclxL and pBad), and downstream of mitochondrial apoptotic pathway (Apaf1, Cytochrome *c* and cleaved-caspase-9) in the cerebral cortices of CON-G, DM-G, and DMEX-G. (**B**) Relative protein quantification of proapoptotic Bcl2-family, pro-survival Bcl2-family and downstream of mitochondrial apoptotic pathway normalized to α-tubulin protein, respectively. Data are expressed as mean ± SD (*n* = 6). * *p* < 0.05, ** *p* < 0.01 compared to CON-G; # *p* < 0.05, ## *p* < 0.01 compared to DM-G. CON-G, control group; DM-G, streptozotocin-induced diabetic group; DMEX-G, streptozotocin-induced diabetes with swimming exercise group.

To identify the neural mitochondria-initiated apoptotic pathway in the diabetic cerebral cortex after swimming training, we detected the components of mitochondria-initiated pathways in CON-G, DM-G, and DMEX-G. Compared with CON-G, the levels of tBid, Bax/Bcl2, Bak/BclxL, Bad/pBad(S136), Apaf1, cytochrome *c*, and cleaved-caspase-9 were significantly increased in DM-G (Figure 3). The expressions of tBid, Bax/Bcl2, Bak/BclxL, Bad/pBad(S136), Apaf1, cytochrome *c*, and cleaved-caspase-9 in DMEX-G were significantly reduced compared with DM-G (Figure 3).

### 3.4. Neural BDNF/TrkB and PI3K/AKT Survival Pathway 

**Figure 4:**
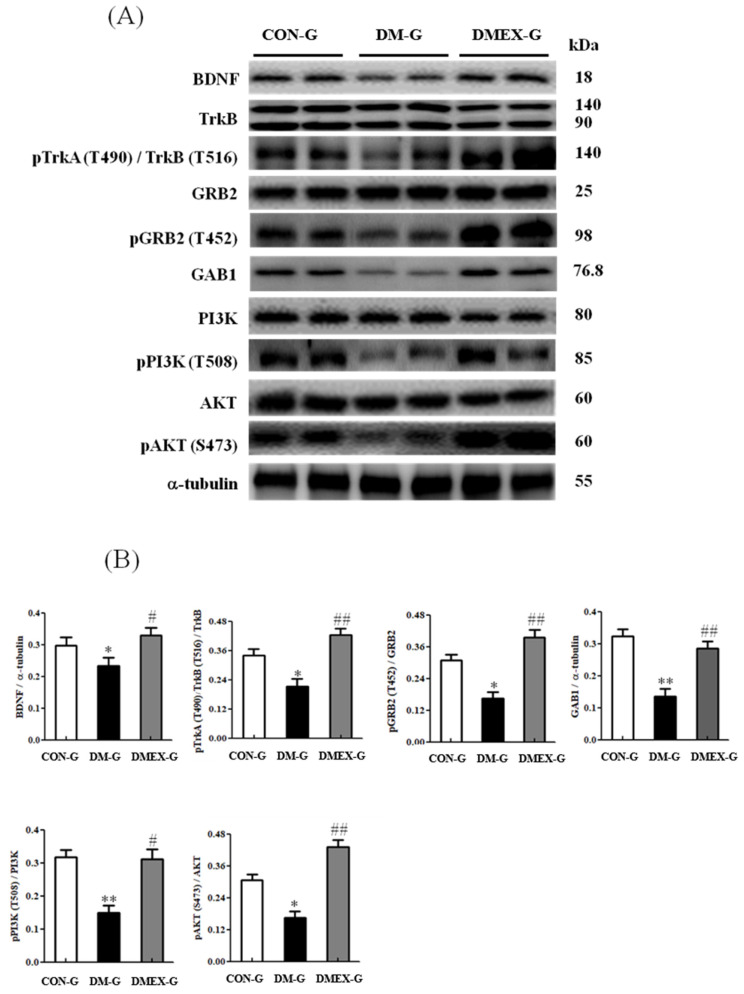
(**A**) Western blot analysis to determine the expressions of BDNF, TrkB, pTrkA (T490)/TrkB (T516), GRB2, pGRB2 (T452), GAB1, PI3K, pPI3K (T508), AKT and pAKT (S473) in the cerebral cortex of CON-G, DM-G and DMEX-G. (**B**) Relative protein quantification of BDNF, TrkB, pTrkA (T490)/TrkB (T516), GRB2, pGRB2 (T452), GAB1, PI3K, pPI3K (T508), AKT and pAKT (S473) normalized to α-tubulin protein, respectively. Data are expressed as mean ± SD (*n* = 6). * *p* < 0.05, ** *p* < 0.01 compared to CON-G; # *p* < 0.05, ## *p* < 0.01 compared to DM-G. CON-G: control group; DM-G: streptozotocin-induced diabetic group; DMEX-G: streptozotocin-induced diabetes with swimming exercise group.

To understand the components of neural BDNF/TrkB and PI3K/AKT survival pathway in the diabetic cerebral cortex after swimming training, we determined the components of the BDNF/TrkB and PI3K/AKT survival pathways in CON-G, DM-G, and DMEX-G. Compared with CON-G, the expressions of BDNF, pTrkA(T490)/TrkB(T516)/TRKB, pGRB2(T452)/GRB2, GAB1, pPI3K(T508)/PI3K, and pAKT(S473)/AKT were significantly decreased in DM-G (Figure 4). The levels of BDNF, pTrkA(T490)/TrkB(T516)/TRKB, pGRB2(T452)/GRB2, GAB1, pPI3K(T508)/PI3K, and pAKT(S473)/AKT in DMEX-G were significantly higher than those in DM-G (Figure 4).

### 3.5. Neural Ras/MEK/MAPK/ERK Signaling Pathway

**Figure 5:**
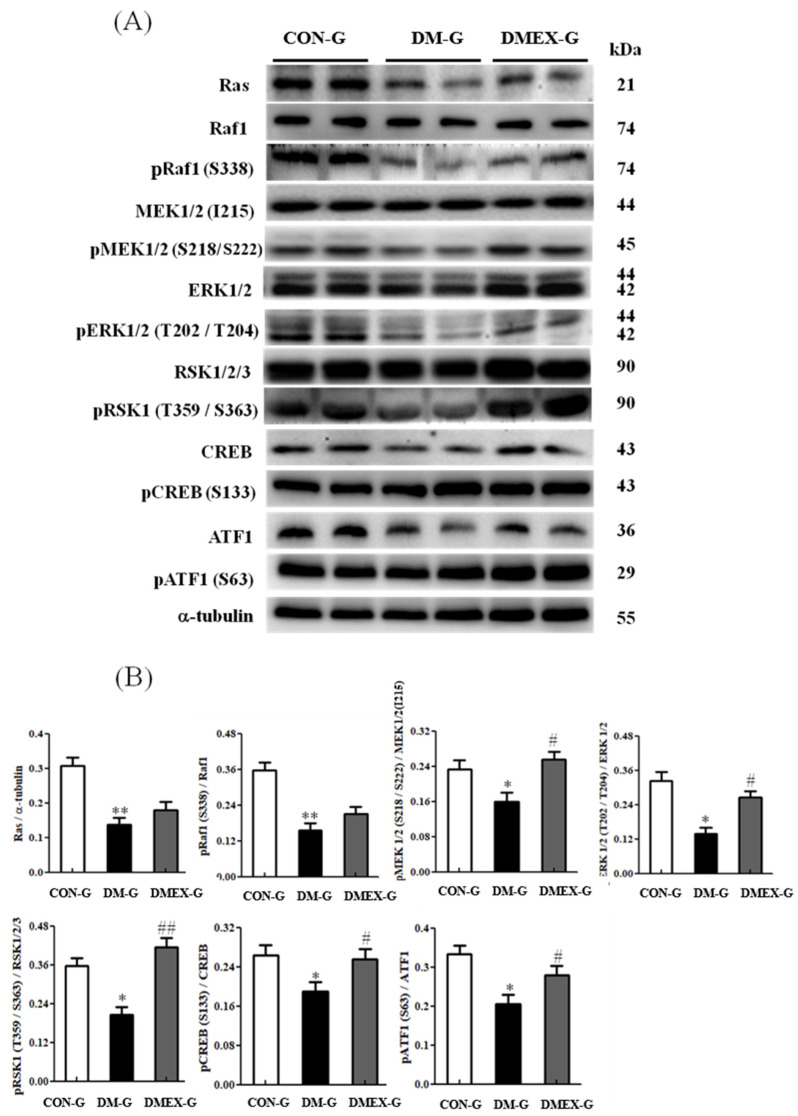
(**A**) Western blot analysis to determine the expressions of Ras, Raf1, pRaf1 (S338), MEK1/2 (I215), pMEK1/2 (S218/222), ERK1/2, pERK1/2 (T202/T204), RSK1/2/3, pRSK1 (T359/S363), CREB, pCREB (S133), ATF1, and pATF1 (S63) in the cerebral cortex of CON-G, DM-G and DMEX-G. (**B**) Relative protein quantification of Ras, Raf1, pRaf1 (S338), MEK1/2 (I215), pMEK1/2 (S218/222), ERK1/2, pERK1/2 (T202/T204), RSK1/2/3, pRSK1 (T359/S363), CREB, pCREB (S133), ATF1, and pATF1 (S63) normalized to α-tubulin protein, respectively. Data are expressed as mean ± SD (*n* = 6). * *p* < 0.05, ** *p* < 0.01 compared to CON-G; # *p* < 0.05, ## *p* < 0.01 compared to DM-G. CON-G: control group; DM-G: streptozotocin-induced diabetic group; DMEX-G: streptozotocin-induced diabetes with swimming exercise group.

To further identify the components of neural Ras/MEK/MAPK/ERK signaling pathway in the diabetic cerebral cortex after swimming training, we detected the components of the Ras/MEK/MAPK/ERK pathways in CON-G, DM-G, and DMEX-G. Compared with CON-G, the levels of Ras, pRaf1/Raf1, pMEK1/2(S218/S222)/MEK1/2(I215), pERK1/2(T202/T204)/ERK1/2, pRSK1(T359/S363)/RSK1/2/3, pCREB(S133)/CREB, and pATF1(S63)/ATF1 were significantly decreased in DM-G (Figure 5). However, the levels of pMEK1/2(S218/S222)/MEK1/2(I215), pERK1/2(T202/T204)/ERK1/2, pRSK1(T359/S363)/RSK1/2/3, pCREB(S133)/CREB, and pATF1(S63)/ATF1 in DMEX-G were significantly increased when compared with DM-G (Figure 5).

## 4. Discussion

**Figure 6:**
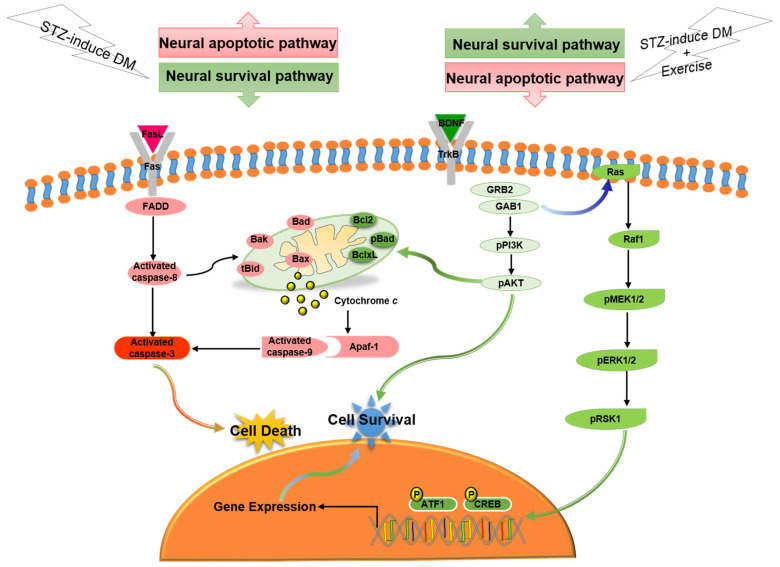
The proposed exercise training can arrest neural cell death in streptozotocin-induced diabetes via suppressing neural Fas/FasL-mediated (FasL, Fas, FADD, cleaved-caspase-8, and cleaved-caspase-3) and mitochondria-initiated (tBid, Bax/Bcl2, Bak/BclxL, Bad, Apaf1, cytochrome *c*, and cleaved-caspase-9) apoptotic pathways as well as enhancing neural BDNF/TrkB survival signaling (BDNF, pTrkB, pGRB2, GAB1, pPI3K, pAKT, Ras, pRaf1, pMEK1/2, pERK1/2, pRSK1, pCREB, and pATF1) and Bcl2-family-mediated survival pathways (Bcl2, BclxL, and pBad).

In the current study, our major findings were: (i) Exercise training reduced diabetes-induced TUNEL(+) apoptotic cells in cerebral cortex. (ii) Exercise training attenuated the diabetes-induced Fas/FasL-mediated apoptotic pathway (FasL, Fas, FADD, cleaved-caspase-8 and cleaved-caspase-3) and attenuated the diabetes-induced mitochondria-initiated apoptotic pathway (Bax, tBid, Bak, Bad, cytosolic cytochrome *c*, cleaved-caspase-9 and cleaved-caspase-3) in the cerebral cortex. (iii) Exercise training enhanced the BDNF/TrkB-mediated PI3K/AKT survival protein levels (BDNF, pTrkB, pGRB2, GAB1, pPI3K, and pAKT), MEK/MAPK/ERK survival protein levels (pMEK1/2, pERK1/2, pRSK1, pCREB and pATF1), and Bcl2-family related survival pathway (pBad, Bcl2, and BclxL) in the diabetic cerebral cortex. Based on our summarized findings, we propose a hypothesized diagram Figure 6 that exercise training not only enhanced neural BDNF/TrkB survival signaling (including the MEK/MAPK/ERK, PI3K/AKT, and Bcl2-family prosurvival pathways), but also prevented diabetes-induced neural Fas/FasL-mediated and mitochondria-initiated apoptotic pathways. 

Exercise is anti-inflammatory action in nature [34,35]. Physical activity leads to reduced inflammatory pathways that improve the development of insulin and leptin resistance, abdominal obesity, atherosclerosis, neurodegeneration, Type 2 diabetes mellitus, dementia, and cardiovascular disease [36]. Consequently, the present STZ-induced diabetic study has to add a cautious note that any beneficial actions of exercise on cerebral cortex changes cannot be isolated to one specific factor or any specific system but may be affected directly or indirectly by various factors, such as hyperglycemia, oxidative stress, inflammatory, endocrine, skeletal muscle produces bioactive molecules (such as interleukin-6, tumor necrosis factor-α) to communicate to other organs (brain, circulatory system, adipose tissue, etc.), or unclear interacting factors.

Apoptosis plays an important role in the progression of neurological disorders [37]. Diabetic encephalopathy or diabetic neurodegeneration is known to damage the central nervous system, which may lead to pathological changes in cerebrovascular and brain structure [3,5,7]. Therefore, we suggested that neural apoptosis might mediate diabetes-induced brain damage. Experimental results have shown that streptozotocin-induced diabetes could significantly increase levels of TUNEL(+) cells, activated caspase-3 [6,8,18,38], Bax [18], Bax/Bcl2, and cytochrome *c* in the hippocampus and cerebral cortex [8]. The present study showed that enlarged interstitial spaces in diabetic cerebral cortex with more neural TUNEL(+) apoptotic cells and cleaved-caspase 3 might change cell morphology. Furthermore, the neural extrinsic apoptotic pathway, including FasL, Fas, FADD, cleaved-caspase-8 and cleaved-caspase 3, and intrinsic apoptotic pathway, including tBid, Bax/Bcl2, Bak/BclxL, Bad/pBad, cytochrome *c*, cleaved-caspase-9 and cleaved-caspase 3, was upregulated in diabetic cerebral cortex in the current study. These findings showed that both neural extrinsic and intrinsic apoptotic pathways are important mechanisms in streptozotocin-induced neural cellular death. Diabetes mellitus is often associated with greater dementia risks and cognitive deficits in the elderly [9,10]. Notably, our previous study indicated that D-galactose-induced aging enhanced neural Fas/FasL-mediated and mitochondria-initiated apoptotic pathways in the cerebral cortex [39]. This is an important report that exercise training attenuated the diabetes-induced Fas/FasL-mediated apoptotic pathway (FasL, Fas, FADD, cleaved-caspase-8, and cleaved-caspase-3) and attenuated the diabetes-induced, mitochondria-initiated, apoptotic pathway (Bax, tBid, Bak, Bad, cytosolic cytochrome *c*, cleaved-caspase-9, and cleaved-caspase-3) in the cerebral cortex, which implies that exercise training could prevent diabetes-induced cerebral cortex neural apoptosis. 

Nitta et al. reported that diabetes attenuated the BDNF expression in brain and caused cognitive impairment [19]. Rozanska et al., suggested that the level and function of BDNF were disturbed by diabetes as well as suggesting that BDNF is a therapeutic target for diabetes [40]. Several studies of streptozotocin-induced diabetic animal model indicated that cognitive decline with downregulation of BDNF and Bcl2 expressions in the hippocampus [18], BDNF and CREB expressions in the hippocampus [30], and BDNF and pTrkB levels in the cerebral cortex [20]. In addition, the impaired PI3K/AKT signaling may contribute to neural apoptosis in the diabetic cerebral cortex in rats induced by streptozotocin [8]. The current study demonstrated that the BDNF/TrkB mediated PI3K/AKT (pGRB2/GRB2, GAB1, pPI3K/PI3K, and pAKT/AKT) and Ras/MEK/MAPK/ERK cascades (Ras, pRaf1/Raf1, pMEK1/2/MEK1/2, pERK1/2/ERK1/2, pRSK1/RSK1/2/3, pCREB/CREB, and pATF1/ATF1) were significantly decreased in the diabetic cerebral cortex. Our data showed that lower BDNF levels caused the downregulation of Ras/MEK/MAPK/ERK and PI3K/AKT survival signaling pathways in the diabetic cerebral cortex. Therefore, we propose that a low level of BDNF may be a risk factor for diabetic neurologic damage. The BDNF/TrkB pathway might play a critical role in the prevention of diabetic neuropathy complications. 

Several studies demonstrated that exercise training or physical exercise improves brain function and elevates brain BDNF levels [25,27,40]. Moreover, previous research has shown the causal link of synapsin I phosphorylation via BDNF, TrkB, and MAP-kinase cascade with downstream facilitated evoked glutamate release [41]. Vaynman et al., reported that the exercise-induced cognitive improvement is dependent on increases in hippocampal BDNF, TrkB, and CREB and synapsin I mRNA levels in sedentary rats, which suggests that exercise induces synaptic plasticity markers in the brain through a BDNF-mediated mechanism by augmenting CREB and synapsin I expression [26]. Furthermore, aerobic treadmill exercise increased significantly the expressions of BDNF, TrkB, and CREB mRNA in the hippocampus and improved spatial memory ability in the streptozotocin-induced diabetic rats [30]. These results provided direct evidence of the potential mechanism of exercise to improve learning and memory by BDNF action to increase neurotransmitter release via phosphorylation of synapsin I and CREB overexpression. In addition, our findings indicated that exercise training prevents neural cell death through upregulating BDNF/TrkB mediated PI3K/AKT (BDNF, pTrkB, pGRB2, GAB1, pPI3K, and pAKT), MEK/MAPK/ERK (pMEK1/2, pERK1/2, pRSK1, pCREB, and pATF1), and Bcl2-family associated prosurvival pathways (pBad, Bcl2, and BclxL). Nevertheless, we found that exercise training did not alter the Ras and pRaf1 levels in diabetic cerebral cortices. In the PLCγ/DAG/IP3 pathway, DAG regulates the production of protein kinase C (PKC), which is required for activation of the MEK/MAPK/ERK cascade [12,17]. Thus, we suggested that the effect of exercise on the MEK/MAPK/ERK cascade could be through the PLCγ/DAG/IP3 signaling. To the best of our knowledge, the present study is the first to elucidate the effects of exercise training on neural extrinsic and intrinsic apoptotic pathways, as well as BDNF/TrkB mediated downstream signaling (including MEK/MAPK/ERK, PI3K/AKT, and Bcl2-family prosurvival pathways) in diabetic cerebral cortices. Enhancing the neural BDNF/TrkB survival pathway via exercise training might be a therapeutic strategy to prevent diabetic-induced neurodegeneration and encephalopathy. Further, neural BDNF/TrkB survival signaling could be a possible hallmark for the beneficial effects in the diabetic brain conferred upon exercise training. 

There were some limitations in the current study. Our animal model by using streptozotocin-induced diabetes for 12 weeks exercise training cannot represent type II diabetes, long-lasting diabetes, or a long-lasting exercise habit. Although the effects of exercise training on extrinsic and intrinsic anti-apoptotic pathways as well as BDNF/TrkB mediated and Bcl2-family pro-survival pathways in diabetic cerebral cortices were positive, we need to make a cautious note that any neuroprotective mechanisms of exercise training on the brain cannot be isolated to one specific factor. Therefore, we should consider various possible direct or indirect mechanisms of exercise training in achieving the anti-apoptotic and pro-survival pathways. 

## 5. Conclusions

These data demonstrated that the neural apoptotic pathway (intrinsic and extrinsic) was activated and BDNF/TrkB survival signaling (MEK/MAPK/ERK, PI3K/AKT, and Bcl2-family survival pathways) were suppressed in the diabetic cerebral cortex. It might provide an important mechanism to explain the development of diabetic encephalopathy. In addition, exercise training enhanced the neural BDNF/TrkB survival pathway and suppressed the neural apoptotic pathway in the diabetic cerebral cortex when considering a novel therapeutic strategy to prevent the development of apoptosis-related neurological diseases in diabetes mellitus. The streptozotocin-induced diabetic animal model under exercise training proves to be a good explanation of clinical exercise training preventing neural apoptosis of the brain in diabetic patients because brain tissues are difficult to extract from diabetic humans. Of course, further investigation is required to elucidate the neuroprotective effects of exercise training in the diabetic human brain. 

## Data Availability

Not applicable.

## References

[1] Zimmet P., Alberti K.G.M.M., Shaw J. (2001). Global and societal implications of the diabetes epidemic. Nature.

[2] Manschot S.M., Biessels G.J., Rutten G.E.H.M., Kessels R.C.P., Gispen W.H., Kappelle L.J. (2008). Peripheral and central neurologic complications in type 2 diabetes mellitus: No association in individual patients. J. Neurol. Sci..

[3] Toth C. (2014). Diabetes and neurodegeneration in the brain. Handb. Clin. Neurol..

[4] Li Z.G., Sima A.A. (2004). C-peptide and central nervous system complications in diabetes. Exp. Diabesity Res..

[5] Moheet A., Mangia S., Seaquist E.R. (2015). Impact of diabetes on cognitive function and brain structure. Ann. N. Y. Acad. Sci..

[6] Wen X., Han X.R., Wang Y.J., Wang S., Shen M., Zhang Z.F., Fan S.H., Shan Q., Wang L., Li M.Q. (2018). Down-regulated long non-coding RNA ANRIL restores the learning and memory abilities and rescues hippocampal pyramidal neurons from apoptosis in streptozotocin-induced diabetic rats via the NF-kappaB signaling pathway. J. Cell. Biochem..

[7] Li Z.G., Zhang W., Sima A.A. (2005). The role of impaired insulin/IGF action in primary diabetic encephalopathy. Brain Res..

[8] Meng Y., Wang W., Kang J., Wang X., Sun L. (2017). Role of the PI3K/AKT signalling pathway in apoptotic cell death in the cerebral cortex of streptozotocin-induced diabetic rats. Exp. Ther. Med..

[9] Bordier L., Doucet J., Boudet J., Bauduceau B. (2014). Update on cognitive decline and dementia in elderly patients with diabetes. Diabetes Metab..

[10] Ninomiya T. (2014). Diabetes mellitus and dementia. Curr. Diabetes Rep..

[11] Lima Giacobbo B., Doorduin J., Klein H.C., Dierckx R., Bromberg E., de Vries E.F.J. (2019). Brain-Derived Neurotrophic Factor in Brain Disorders: Focus on Neuroinflammation. Mol. Neurobiol..

[12] Bathina S., Das U.N. (2015). Brain-derived neurotrophic factor and its clinical implications. Arch. Med. Sci..

[13] Huang E.J., Reichardt L.F. (2001). Neurotrophins: Roles in neuronal development and function. Annu. Rev. Neurosci..

[14] Maisonpierre P.C., Le Beau M.M., Espinosa R., Ip N.Y., Belluscio L., de la Monte S.M., Squinto S., Furth M.E., Yancopoulos G.D. (1991). Human and rat brain-derived neurotrophic factor and neurotrophin-3: Gene structures, distributions, and chromosomal localizations. Genomics.

[15] Noble E.E., Billington C.J., Kotz C.M., Wang C. (2011). The lighter side of BDNF. Am. J. Physiol. Regul. Integr. Comp. Physiol..

[16] Skaper S.D. (2008). The biology of neurotrophins, signalling pathways, and functional peptide mimetics of neurotrophins and their receptors. CNS Neurol. Disord.-Drug Targets.

[17] Patapoutian A., Reichardt L.F. (2001). Trk receptors: Mediators of neurotrophin action. Curr. Opin. Neurobiol..

[18] Tian Z., Wang J., Xu M., Wang Y., Zhang M., Zhou Y. (2016). Resveratrol Improves Cognitive Impairment by Regulating Apoptosis and Synaptic Plasticity in Streptozotocin-Induced Diabetic Rats. Cell. Physiol. Biochem..

[19] Nitta A., Murai R., Suzuki N., Ito H., Nomoto H., Katoh G., Furukawa Y., Furukawa S. (2002). Diabetic neuropathies in brain are induced by deficiency of BDNF. Neurotoxicol. Teratol..

[20] Liu P., Li H., Wang Y., Su X., Li Y., Yan M., Ma L., Che H. (2020). Harmine Ameliorates Cognitive Impairment by Inhibiting NLRP3 Inflammasome Activation and Enhancing the BDNF/TrkB Signaling Pathway in STZ-Induced Diabetic Rats. Front. Pharmcol..

[21] Chan C.B., Ahuja P., Ye K. (2019). Developing Insulin and BDNF Mimetics for Diabetes Therapy. Curr. Top. Med. Chem..

[22] Zhen Y.F., Zhang J., Liu X.Y., Fang H., Tian L.B., Zhou D.H., Kosten T.R., Zhang X.Y. (2013). Low BDNF is associated with cognitive deficits in patients with type 2 diabetes. Psychopharmacology.

[23] Krabbe K.S., Nielsen A.R., Krogh-Madsen R., Plomgaard P., Rasmussen P., Erikstrup C., Fischer C.P., Lindegaard B., Petersen A.M., Taudorf S. (2007). Brain-derived neurotrophic factor (BDNF) and type 2 diabetes. Diabetologia.

[24] Ferris L.T., Williams J.S., Shen C.L. (2007). The effect of acute exercise on serum brain-derived neurotrophic factor levels and cognitive function. Med. Sci. Sports Exerc..

[25] Seifert T., Brassard P., Wissenberg M., Rasmussen P., Nordby P., Stallknecht B., Adser H., Jakobsen A.H., Pilegaard H., Nielsen H.B. (2010). Endurance training enhances BDNF release from the human brain. Am. J. Physiol.-Regul. Integr. Comp. Physiol..

[26] Vaynman S., Ying Z., Gomez-Pinilla F. (2004). Hippocampal BDNF mediates the efficacy of exercise on synaptic plasticity and cognition. Eur. J. Neurosci..

[27] Rasmussen P., Brassard P., Adser H., Pedersen M.V., Leick L., Hart E., Secher N.H., Pedersen B.K., Pilegaard H. (2009). Evidence for a release of brain-derived neurotrophic factor from the brain during exercise. Exp. Physiol..

[28] Lang X., Zhao N., He Q., Li X., Sun C., Zhang X. (2020). Treadmill exercise mitigates neuroinflammation and increases BDNF via activation of SIRT1 signaling in a mouse model of T2DM. Brain Res. Bull..

[29] Żebrowska A., Hall B., Maszczyk A., Banas R., Urban J. (2018). Brain-derived neurotrophic factor, insulin like growth factor-1 and inflammatory cytokine responses to continuous and intermittent exercise in patients with type 1 diabetes. Diabetes Res. Clin. Pract..

[30] Tang L., Kang Y.T., Yin B., Sun L.J., Fan X.S. (2017). Effects of weight-bearing ladder and aerobic treadmill exercise on learning and memory ability of diabetic rats and its mechanism. Chin. J. Appl. Physiol..

[31] Lay I.S., Kuo W.W., Shibu M.A., Ho T.J., Cheng S.M., Day C.H., Ban B., Wang S., Li Q., Huang C.Y. (2021). Exercise training restores IGFIR survival signaling in d-galactose induced-aging rats to suppress cardiac apoptosis. J. Adv. Res..

[32] Cheng S.M., Ho T.J., Yang A.L., Chen I.J., Kao C.L., Wu F.N., Lin J.A., Kuo C.H., Ou H.C., Huang C.Y. (2013). Exercise training enhances cardiac IGFI-R/PI3K/Akt and Bcl-2 family associated pro-survival pathways in streptozotocin-induced diabetic rats. Int. J. Cardiol..

[33] Cheng S.M., Cheng Y.J., Wu L.Y., Kuo C.H., Lee Y.S., Wu M.C., Huang C.Y., Ting H., Lee S.D. (2014). Activated apoptotic and anti-survival effects on rat hearts with fructose induced metabolic syndrome. Cell Biochem. Funct..

[34] de Lemos E.T., Reis F., Baptista S., Pinto R., Sepodes B., Vala H., Rocha-Pereira P., da Silva G.C., Teixeira N., Silva A.S. (2009). Exercise training decreases proinflammatory profile in Zucker diabetic (type 2) fatty rats. Nutrition.

[35] Das U.N. (2004). Anti-inflammatory nature of exercise. Nutrition.

[36] UN D., Farooqui T., Farooqui A.A. (2015). Molecular, biochemical, and physiological basis of beneficial actions of exercise. Diet and Exercise in Cognitive Function and Neurological Diseases.

[37] Mattson M.P. (2000). Apoptosis in neurodegenerative disorders. Nat. Rev. Mol. Cell Biol..

[38] Liu J., Feng L., Ma D., Zhang M., Gu J., Wang S., Fu Q., Song Y., Lan Z., Qu R. (2013). Neuroprotective effect of paeonol on cognition deficits of diabetic encephalopathy in streptozotocin-induced diabetic rat. Neurosci. Lett..

[39] Cheng S.M., Ho Y.J., Yu S.H., Liu Y.F., Lin Y.Y., Huang C.Y., Ou H.C., Huang H.L., Lee S.D. (2020). Anti-Apoptotic Effects of Diosgenin in D-Galactose-Induced Aging Brain. Am. J. Chin. Med..

[40] Rozanska O., Uruska A., Zozulinska-Ziolkiewicz D. (2020). Brain-Derived Neurotrophic Factor and Diabetes. Int. J. Mol. Sci..

[41] Jovanovic J.N., Czernik A.J., Fienberg A.A., Greengard P., Sihra T.S. (2000). Synapsins as mediators of BDNF-enhanced neurotransmitter release. Nat. Neurosci..

